# Supplementary material for: ADP binding by the Culex quinquefasciatus mosquito D7 salivary protein enhances blood feeding on mammals
Source: Nat Commun. 2020 Jun 9;11:2911. doi: 10.1038/s41467-020-16665-z (PMC7283271; doi:10.1038/s41467-020-16665-z)
Supplement: Supplementary file 1 — Supplementary Information [file 41467_2020_16665_MOESM1_ESM.pdf]

Supplementary Information

ADP binding by the *Culex quinquefasciatus* mosquito D7 salivary protein enhances blood feeding on mammals

Martin-Martin *et al.*

Supplementary Figures

```
CxD7L1       1 DEWS-PMDPEEVAFEEAKCMEDHFGNDF---GLAEKWMKWSLAESDG-KTACYVKCLVEA
CxD7L2       1 -AWK-PFSPEETLFTYTRCMEDNAKGDL---ALAKKWMAWKLE-ADQ-KSACYAKCVLVG
AeD7L1       1 --MG-PFDPEEMLFIFTRCMEDNLEDGANRLPMLAKWKEWINEPVDSPATQCFGKCVLVR
AnStD7L1     1 QPWK-ALDAEQALYVYKRCYEDHLPSGSDRKTYMTLWNAWRLEPNDA-ITHCYAKCVLTG
PhDubD7L     1 --WRYPRNADQTFWAFRTCQRQSEGA-----KSLREWYRWNLP-NDE-DTHCYVKCVWLH

CxD7L1      56 LGMYDKQ--AFQPNNIKQQYEAYKSDNGVDQTK---GDAIANELG-KIDAKDGKCESIAK
CxD7L2      54 LELFDESSKTFKGDHILEQYQKYKSYTSQDEAGVKKFQQAVQALG-TIDS--ADCLKVLQ
AeD7L1      58 TGLYDPVAQKFDASVIQEQFKAYPSLGEKSK--V---EAYANAVK-QLPSTNNDCAAVFK
AnStD7L1    59 LQIYDPQENAFKSDRIPVQYQAYKTITQSKQKEVTEYQ---KALA-AANAKSGSCVDLYN
PhDubD7L    52 LGLYNEQNKSLRVDRIMEQFNSRSVAIP-------------GGINTISGPTDGTCKDIYD

CxD7L1     110 GFIQVNNANKGVLEKIYLLDSSVRDAIYKKN-PQIKPKGISIFRFCGKQFYQDGE---AA
CxD7L2     111 KYGPVHAQFTDVQRNVYFGKKEITDKIYNSD-STVKKRDETMFRFCERSNFKDGS---EE
AeD7L1     112 AYDPVHKAHKDTSKNLFHGNKELTKGLYEKLGKDIRQKKQSYFEFCENKYYPAGSDKRQQ
AnStD7L1   115 AYLPVHNRFVNLSRQLYHGTVEGAAKIYAAM-PEIKQKGESFHAYCEKRAWKGNK--QSE
PhDubD7L    99 KTINFFNNNVNDLRTAFYGIKKLSDEWFTQN-SNTKPKGTKISDFCNAENREKGGADCQH

CxD7L1     166 YCNVRKHGFSDDPKFIKHSNCTTRGMRWMKKNGEMDESAILRGLHAVNENGKDDVVKKSL
CxD7L2     167 LCTLRKTGITTNN---NHLDCLFRGLRYLDRNGNINPDEIKRDLHFINVKDKDAAVDNAL
AeD7L1     172 LCQIRQYTVLDDALFKEHTDCVMKGIRYITKDNQLDVEEVKRDFKLVNKDTK--ALEEVL
AnStD7L1   172 WKNGRRYKLTGSPELKDAIDCIFRGLRYMDDTG-LKVDEIVRDFNLINKSELEPEVRSVL
PhDubD7L   158 ACSAYYYRLVDEDNE----PIHFRNLNILGITDEQFASCVK-----A---------SKNK

CxD7L1     226 QNCKAKDES----KARDYYKCIYDGLG-EQLFMKVLDYIEVRSENYSYRLREATSKYDAN
CxD7L2     224 NNCKVKEAT----KATDYNDCLWKDPNLKDIMMPVFDYREVRSESYRYFIE-NTDPYDVA
AeD7L1     230 NDCKSKEPSNAKEKSWHYYKCLVES-SVKDDFKEAFDYREVRSQIYAFNLPKNQA-YSKP
AnStD7L1   231 ASCKGS-------EAYDYYVCLVNS-RLKQHFKNAFDFHELRSADYAYLLRGKVY-ENPE
PhDubD7L   200 QGCKV---------ADTMYNCVEKHN------SQALKILDNQSPTY--------------

CxD7L1     281 AMRSKVKALDSEAKC-
CxD7L2     279 KVKEKVKKYDKDAGC-
AeD7L1     288 AVQSQVMEIDGKQCPQ
AnStD7L1   282 KVKEEMKKLNTTVHF-
PhDubD7L       ----------------
```

Supplementary Fig. 1. Alignment of *C. quinquefasciatus* D7 proteins and related sequences.

Comparison of *Culex* D7 long proteins: CxD7L1 (AAL16046) and CxD7L2 (AAL16047) with *Ae. aegypti* D7: AeD7 (PDB ID: 3DZT), *An. stephensi* D7L1: AnStD7L1 (PDB ID: 3NHT), and *Phlebotomus duboscqi* PhDubD7L (PDB ID: 6MT7). Sequences without a signal peptide were aligned with Clustal Omega (EMBL) and refined using BoxShade server version 3.1 (Expasy Bioinformatics Resource Portal).

Black background shading represents amino acids involved in the eicosanoid binding of AeD7, AnStD7L1, and PhDubD7L[1-3]. Magenta shading highlights amino acids involved in biogenic amine binding for AeD7[2]. Cyan shading indicates amino acids involved in ADP binding for CxD7L1. Position K52, highlighted with an arrow, is involved in TXA_2 binding[1]. Gray shading shows conserved residues of the amino acids involved in ligands binding.

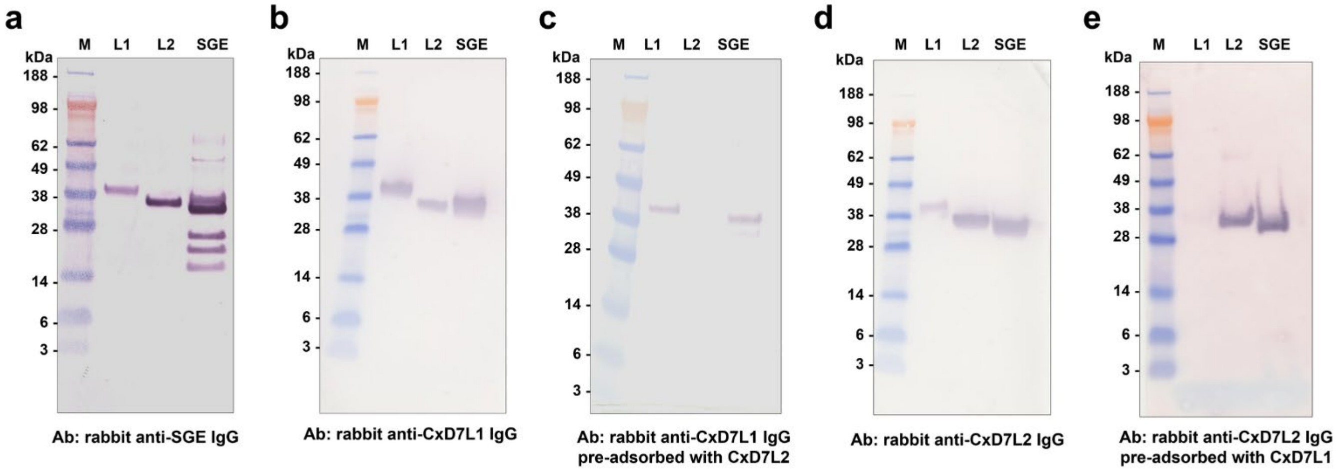

Supplementary Fig. 2: Western blot analysis of recombinant CxD7L1 and CxD7L2.

(a) Purified IgG from serum of a rabbit immunized with salivary gland extract (SGE) from *Culex quinquefasciatus* recognized the recombinant proteins CxD7L1 and CXD7L2 (100 ng) and other protein bands from the salivary gland extract (2.5 µg). **(b)** Purified IgG from serum of a rabbit immunized with CxD7L1 protein recognized CxD7L1 recombinant protein (100 ng) and a band of similar molecular weight in the SGE (2.5 µg). It also cross-reacted with CxD7L2. **(c)** Purified IgG from serum of a rabbit immunized with CxD7L1 protein and pre-adsorbed with CxD7L2 specifically recognized CxD7L1 recombinant protein (100 ng) and a band of similar molecular weight in the SGE (2.5 µg). **(d)** Purified IgG from serum of a rabbit immunized with CxD7L2 protein recognized CxD7L2 recombinant protein (100 ng) and a band of similar molecular weight in SGE (2.5 µg). It also cross-reacted with CxD7L1 **(e)** Purified IgG from serum of a rabbit immunized with CxD7L2 protein and pre-adsorbed with CxD7L1 specifically recognized CxD7L2 recombinant protein (100 ng) and a band of similar molecular weight in the SGE (2.5 µg). No cross-reactivity between anti-CxD7L1 IgG and anti-CxD7L2 IgG was observed after anti-CxD7L1 was pre-adsorbed with CxD7L2 and anti-CxD7L2 was pre-adsorbed with CxD7L1. Anti-Culex SGE IgG antibodies were used at 1 µg/ml and IgG antibodies against recombinant proteins were used at 0.5 µg/ml. Goat anti-rabbit IgG AP (1:10,000 dilution, Sigma) was used as a secondary antibody. Western blots shown are representative of two independent experiments. SeeBlue Plus2 Pre-stained was used as the protein standard (M). Source data are provided as a Source Data file.

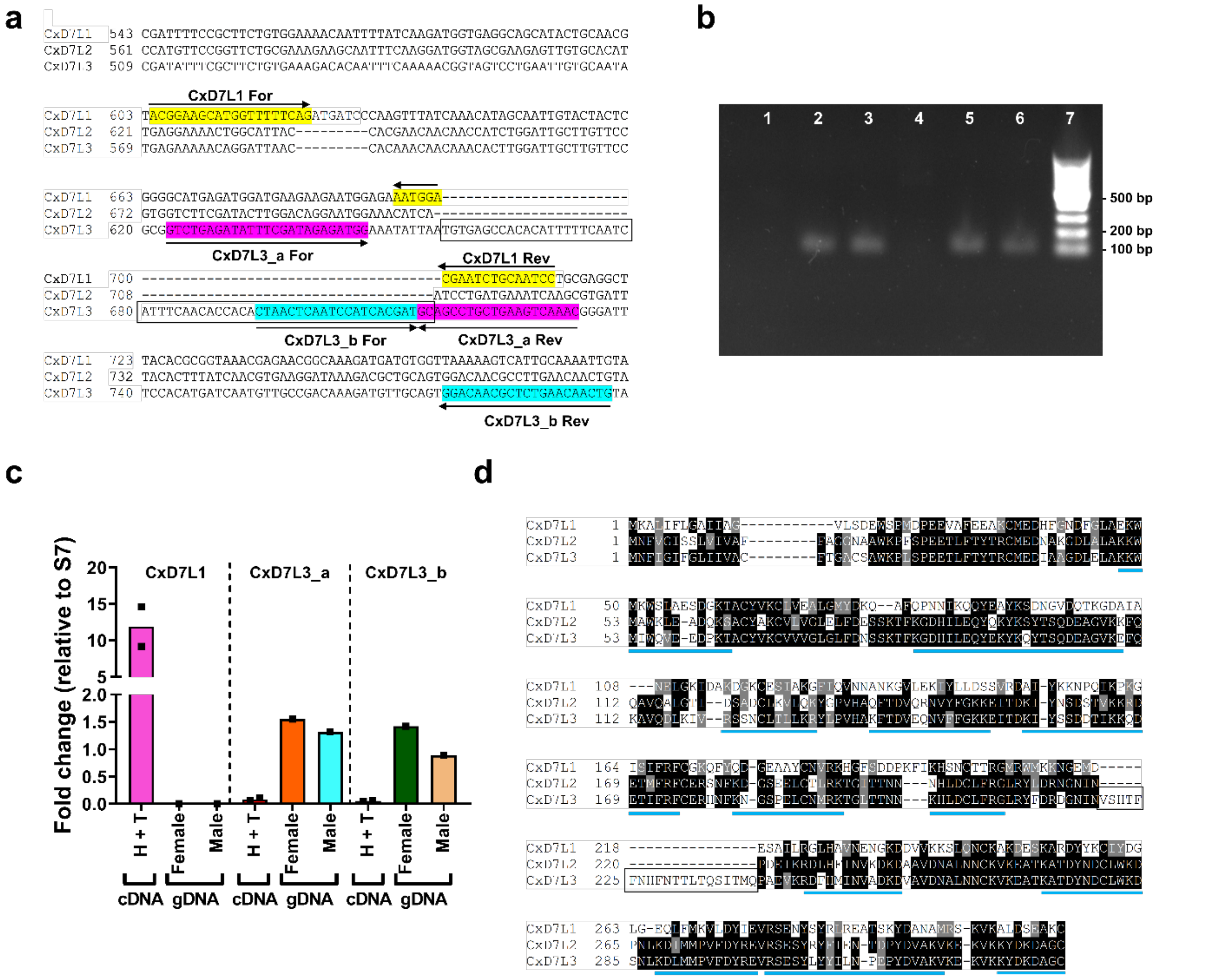

Supplementary Fig. 3: CxD7L3 is not expressed in salivary glands of *C. quinquefasciatus* (a) Alignment of DNA sequences of CxD7L1 (AF420269), CxD7L2 (AF420270), and CxD7L3 (AY388553). Specific primers for CxD7L1 are shown in yellow (CxD7L1 For and CxD7L1 Rev) and CxD7L3 primers are highlighted in magenta (CxD7L3_a For and CxD7L3_a Rev) and cyan (CxD7L3_b For and CxD7L3_b Rev). CxD7L3 nucleotide unique insertion is boxed. **(b)** Sybr Safe 1.2% agarose gel electrophoresis (n = 1) of Phire PCR products using CxD7L3 primer sets a and b are shown in lanes 1-3 and 4-6, respectively. gDNA extracted from one leg of female (lanes 2 and 5) and male (lanes 3 and 6) mosquito adults were used as DNA templates for PCR. Reaction mixes without DNA were included as negative controls (lanes 1 and 4). GeneRuler 100 bp DNA ladder was used (Thermo Scientific). **(c)** Gene

expression analysis of *CxD7L1* and *CxD7L3* transcripts using cDNA from head and thorax from female mosquitoes and gDNA from female and male adult mosquitoes. Relative abundance was expressed as the fold change using the 40S ribosomal protein S7 as the housekeeping gene. *CxD7L1* was highly expressed in the head and thorax of the mosquitoes (n= 2), but not present in our gDNA samples (n = 1). However, *CxD7L3* was equally present in female and male gDNA and absent in cDNA. Both primer sets CxD7L3_a and b showed similar results. Data are presented as mean values. **(d)** Amino acid sequence alignment of CxD7L1 (AAL16046), CxD7L2 (AAL16047), and CxD7L3 (AAR18441). Peptides identified by mass spectrometry in SGE of *C. quinquefasciatus* are underlined in blue. Specific CxD7L3 amino acid unique insertion is boxed. Mass spectrometry was done with biological triplicates of SGE from 10 female mosquitoes each. Source data are provided as a Source Data file.

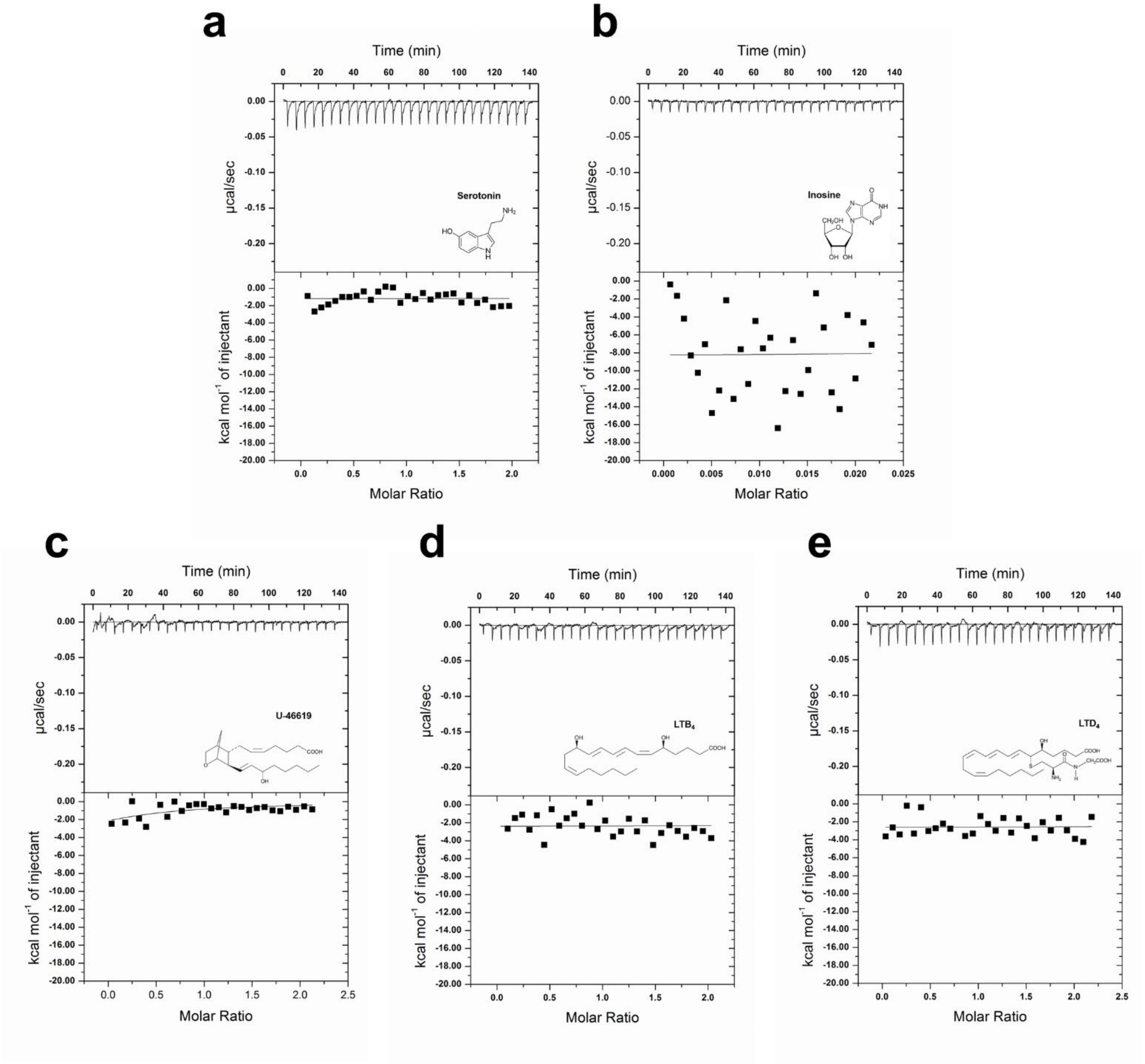

Supplementary Fig. 4. Isothermal titration calorimetry studies of CxD7L1.

Binding experiments were performed on a VP-ITC microcalorimeter. **(a)** 30 μM serotonin or **(b)** 30 μM inosine were titrated with 3 μM of CxD7L1. For TXA_2 analog U-46619 **(c)** and leukotrienes LTB_4 **(d)** and LTD_4 **(e)** protein and ligand were prepared at 5 μM and 50 μM, respectively. Assays were performed at 30 °C. The upper curve in each panel shows the measured heat for each injection, while the lower graph shows the enthalpies for each injection and the fit to a single-site binding model for calculations of

thermodynamic parameters. Titration curves are representative of two measurements. The insets show the names and chemical formulas for these compounds.

```
CxD7L1_AAL16046.1    1 -----MK---ALI-FLGAI------------------IAGVLSDEWSPMDPEEVAFEEAKC
Ctar_JAV19160.1      1 ---MNSRVVAVLVTVLVQAVCIWSAAVPACPDDSSQPAAADPSSWTPRNPEQTMYAYVRC
Ctar_JAV18819.1      1 ---------SKIL-SLIQN-----------------KCPNQITAWKPLDPEQVLFGFSRC
Ctar_JAV29109.1      1 MDKMPTVFVTALL-LLIS------------------VNQSLTLASLAFDPEETRFVLTRC
Ctar_JAV19100.1      1 -----MNLISITG-LLIVA-----------------FFTGGNAQWKPFNPEETLFTYTRC
Ctar_JAV19156.1      1 ------M---IKI-VVLLA-----------------FGGIVSGQWSPMNPEEVAFEQAKC

CxD7L1_AAL16046.1   35 MEDHFGND-------FGLAEKWMKWSLAES--DGKTACYVKCLVEALGMYDKQ--AFQPN
Ctar_JAV19160.1     58 LNDSTAS--------VEQKIRWVRWQPDAS---TESQCYVKCVSEELRLFDVHERRFRPE
Ctar_JAV18819.1     34 GEDH----TPNDENRTIRIQNWAQWKLEPV--DNWTMCYVQCCLEKLGLFNVTTKKFMTD
Ctar_JAV29109.1     42 IEQYSTPSVDDDSSRQARIRDWISWKLDAAAGDEQTKCFVACLLNKLKLWQPYLGKFRGE
Ctar_JAV19100.1     38 MEDNAKGD-------MELAKKWMNWKLK-Q--DPKSACYAKCVLVGLELFDESSKTFKGD
Ctar_JAV19156.1     34 IEDHFKDD-------FTVAEQWLDWKLAKG--DPKTPCYVKCLAEALGIYDDQAKAFQPN

CxD7L1_AAL16046.1   84 NIKQQYEAYKSDNGVDQTKGDAIANELGKID-AKDGKCESIAKGFIQVNNANKGVLEKIY
Ctar_JAV19160.1    107 RFVLQAETYGRGDVNGELDKLRTNAKPMLAGSLEEVTCETVFNKYATFYATHTETILKMF
Ctar_JAV18819.1     88 HLNSQYEGFKKYNELNLTQVNEFATALNSFGEL--HSCADVFRALTVELKAHMLTLIKLF
Ctar_JAV29109.1    102 QLLLQHDLYNSYVNWSRADVEEFARAVEQTGDV--WNCQAVYEGFKVAILPQMVMFKQLF
Ctar_JAV19100.1     88 HILEQYQKYKSYTTQDEAGVKEFQNAVQALGSVESSDCLKVLQKYAPVHAKYTDVQRNVY
Ctar_JAV19156.1     85 NINQQYEAYKGDNGVEPAKAEAIQKELEKID-VKDGKCESIGRGILKVESANQGILKKIY

CxD7L1_AAL16046.1  143 LLDSSVRDAIYKK-NPQIKPKGISIFRFCGKQFYQDG-EAAYCNVRKHG-FSDDPKFIKH
Ctar_JAV18819.1    146 NGSPRINTKIYEDLGPTIRQRKQSYVEFCENLFLKDN-KIIVCNFRLRRKQRTDGHYKQL
Ctar_JAV29109.1    160 LLEDEIAGNIYADLGTSIRQPNQSYFQFCEKRYYRNQ-VDIWCTARNYS-IPDDRNFHKH
Ctar_JAV19100.1    148 FGKKEITDKIYST-DSTVKKRDETMFRFCERSNFQDG-SAELCTLRKTG-ITTKN---EH
Ctar_JAV19160.1    167 HGDHRDLMETYGKLGDKVKQIGETFVAYCEKRYGGSWNEDEACP------------ATAL
Ctar_JAV19156.1    144 LIDSAVKDAIYKK-NPQIKPKGVSIFRFCGKQFYTDG-EPAYCNVRRHG-YSDDEKFIRH

CxD7L1_AAL16046.1  200 SNCTTRGMRWMKKNGEMDESAILRGLHAVNENGKDDVVKKSLQNCKAKDE-SKARDYYKC
Ctar_JAV19160.1    215 VDCVLRGFRWITEEGEVNVNEIRRDYAAAGFSDSDEASCTS------A---AGARDLFQC
Ctar_JAV18819.1    205 IDCIFKGFRYLDKEEKLDAEEIIRDFHAIGKTKLDDDIQMTLTNCFKESQPSTAQNYYDC
Ctar_JAV29109.1    218 MDCIFRGLRYFDRDEALNVVEILRDFHLAEVTNLDDEITNSLVLCEVESG-SEALSYYRC
Ctar_JAV19100.1    202 LDCLFRGLRYMDRNGNINPAEIKRDLHFINVNDKDDAVDNALNNCKVNEA-TKATDYNDC
Ctar_JAV19156.1    201 SNCTTRGMRWMKKNGEMDESAILRDLHAVEENSKDDVVKSSLQNCKAKDE-TKARDYYKC

CxD7L1_AAL16046.1  259 IYDGLG--EQLFMKVLDYIEVRSENYSYRLREATS-KYDANAMRSKVKALDSEAKC----
Ctar_JAV19160.1    266 LRGLTADGATRLNQVIRERNQRTAFYFDATSQEEP-WRSAVEFGQQRMNL----------
Ctar_JAV18819.1    265 LMSSEKL-EKPFQAAFDYREFRSSDYDYAFAIPGPPIYDCHQVAAAKRKRISALDCN---
Ctar_JAV29109.1    277 LLDSNFV--EQFKDALDYREIRSIDYFHRLRDSVP-SYNRDEIHQ-KVNEIHRNYCIMAK
Ctar_JAV19100.1    261 LWKDPNL-KDIMMPVFDYREVRSESYRYFIE-HTE-PYDATKVKEKVRKYDQDAGC----
Ctar_JAV19156.1    260 IYDGLG--EQLFMKVLDYVEVRSENYGFRLRKETS-KYDPSAMRTKVQDLDTAAKCPPVA

CxD7L1_AAL16046.1      -----
Ctar_JAV19160.1        -----
Ctar_JAV18819.1        -----
Ctar_JAV29109.1        -----
Ctar_JAV19100.1        -----
Ctar_JAV19156.1    317 HGSKK
```

Supplementary Fig. 5. Comparison of CxD7L1 with *Culex tarsalis* D7 long proteins. Eighteen D7 homologs from *C. tarsalis* were retrieved from NCBI database after a tBLAST search using the CxD7L1

protein as the query sequence. The database used was the Transcriptome Shotgun Assembly, BioProject PRJNA360148. *Culex tarsalis s*equences with E value lower than 4e-10 were chosen (N = 10) and clustered by cd-hit software[4] where sequence identity cut-off was set at 0.85. CxD7L1 (AAL16046) and 5 representative of *C. tarsalis* D7 long protein homologs (JAV19160, JAV18819, JAV29109, JAV19100, and JAV19156) were aligned with Clustal Omega (EMBL) and refined using BoxShade server version 3.1 (Expasy Bioinformatics Resource Portal). Black background shading represents identical amino acids (50% of sequences must agree for shading) while grey shading designates similar amino acids. Residues highlighted in blue indicate conserved amino acids involved in ADP binding.

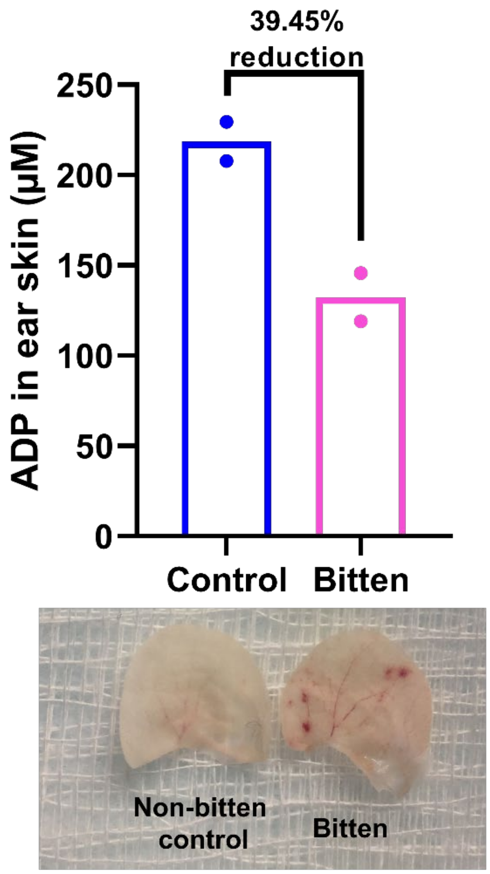

Supplementary Fig. 6. ***Culex quinquefasciatus*** **bites reduced ADP levels in mouse ears.**

ADP levels were determined in ears of Balb/c mice bitten by *C. quinquefasciatus* mosquitoes. For each animal, the right ear was bitten by 5-10 mosquitoes while the left ear was used as a non-bitten control. Immediately after mosquito blood-feeding, 3 mm diameter biopsies were taken from the bite sites or control skin, and samples were processed for ADP determination (Abcam, Cat no. ab211087) in the presence of protease inhibitors. Salivary proteins with bound ADP were separated from unbound ADP by using 10 kDa Amicon ultra centrifugal filters (Millipore). Results were interpolated from a standard ADP curve as pmol/sample. The volume of the ear plug was calculated (1.59 µl) as the radius of the skin cylinder (1.5 mm) multiplied by the skin height (normalized as 0.225 mm)[5]. ADP results were expressed as the concentration of ADP in skin (µM). Two mice were used in this experiment. Data are presented as mean values. Source data are provided as a Source Data file.

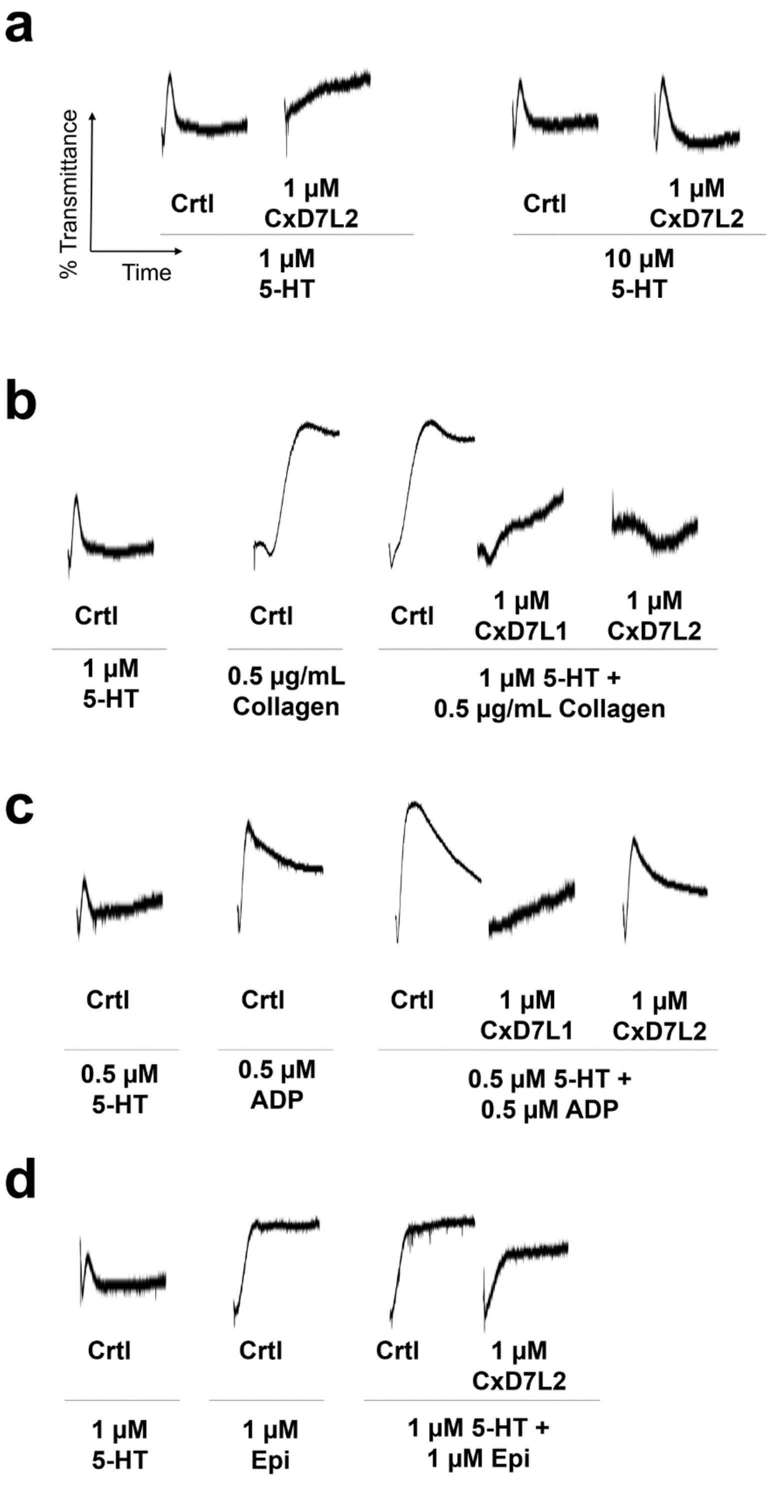

Supplementary Fig. 7. Effect of CxD7L1 and CxD7L2 on platelet aggregation. Prior to the addition of the agonist, platelet-rich human plasma was incubated for 1 minute either with PBS (Crtl) or with the

recombinant proteins at the concentrations shown. Aggregometer traces were measured at 37 °C from stirred platelets suspensions on a Chrono-Log platelet aggregometer model 700 for 6 min. An increase of light transmittance over time indicates platelet aggregation. **(a)** Platelet aggregation traces using different concentrations of serotonin (5-HT) (1 μM and 10 μM) as aggregation agonist. **(b)** Platelet aggregation traces using 5-HT in combination with collagen, **(c)** ADP or **(d)** epinephrine (Epi). Graphs are representative of two measurements. Source data are provided as a Source Data file.

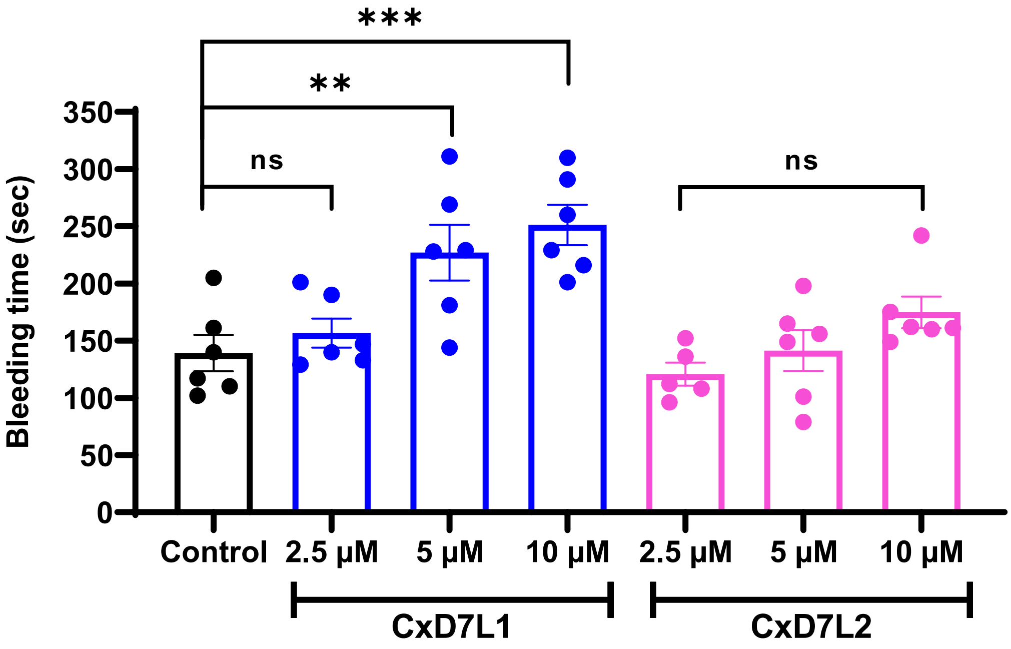

Supplementary Fig 8. CxD7L1 and CxD7L2 impair host hemostasis *in vivo*. The mice tails were transected at 1 cm from the tip and placed in tubes containing either saline (control) or CxD7L1 and CxD7L2 recombinant proteins at the concentrations indicated in the figure. The time from the incision to the cessation of bleeding was recorded as the tail vein bleeding time. Results from six animals are represented for all groups, except the group of 2.5 µM of CxD7L2 that included five animals. Multiple comparisons were carried out by one-way ANOVA with Dunnett's correction (***: $p = 0.0002$; **: $p = 0.0038$; ns: not significant). Data are presented as mean values +/- SEM. Source data are provided as a Source Data file.

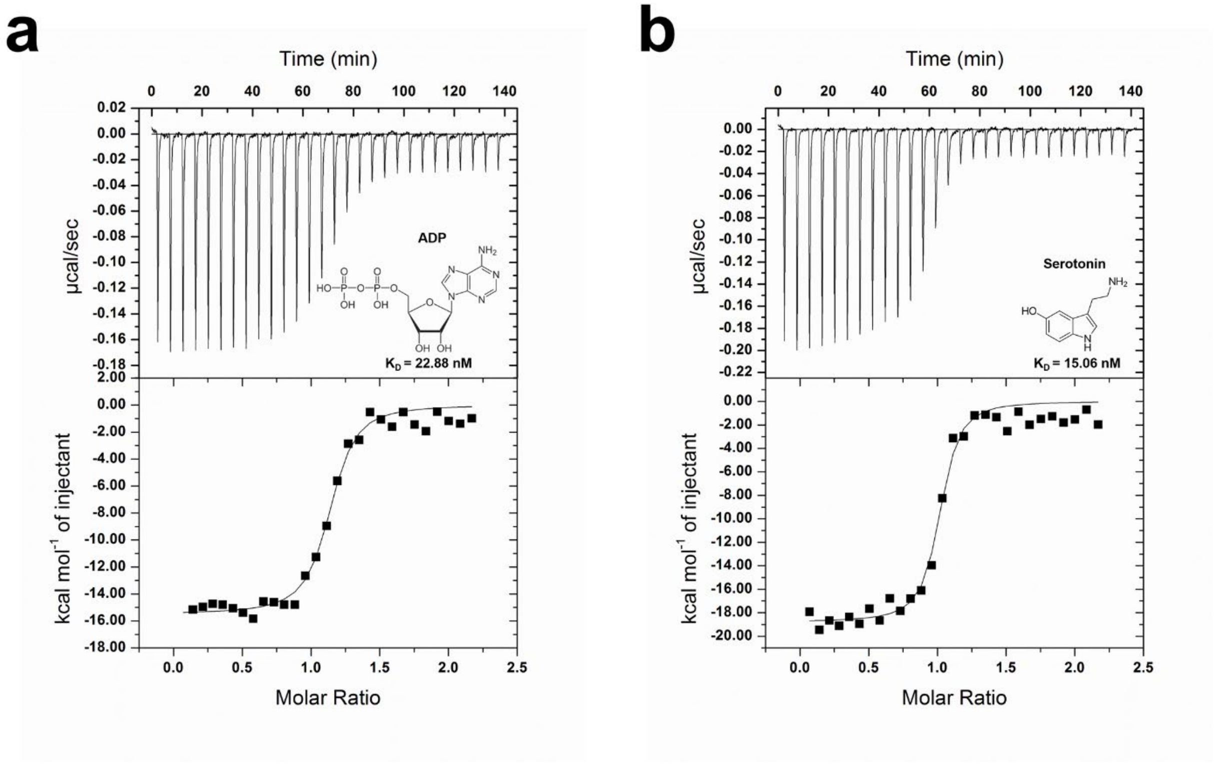

Supplementary Fig. 9. Anti-CxD7 antibodies do not block ligand binding capacity. Both protein and ligand solutions were incubated with their correspondent antibodies at a final concentration of 6 µM for 30 min at 4 °C. Binding experiments were performed on a VP-ITC microcalorimeter. Assays were performed at 30 °C. The upper curve in each panel shows the measured heat for each injection, while the lower graph shows the enthalpies for each injection and the fit to a single-site binding model for calculation of thermodynamic parameters. Titration curves are representative of two measurements. Panel a shows CxD7L1 binding to ADP in the presence of 6 µM rabbit anti-CxD7L1 IgG. Panel b shows CxD7L2 binding to serotonin in the presence of 6 µM rabbit anti-CxD7L2 IgG. The insets show the names and chemical formulas for these compounds.

Supplementary References

1. Alvarenga, P.H.*, et al.* The function and three-dimensional structure of a thromboxane A2/cysteinyl leukotriene-binding protein from the saliva of a mosquito vector of the malaria parasite. *PLoS Biol.* **8**, e1000547 (2010).
2. Calvo, E., Mans, B.J., Ribeiro, J.M. & Andersen, J.F. Multifunctionality and mechanism of ligand binding in a mosquito antiinflammatory protein. *Proc. Natl. Acad. Sci. U S A* **106**, 3728-3733 (2009).
3. Jablonka, W.*, et al.* Functional and structural similarities of D7 proteins in the independently-evolved salivary secretions of sand flies and mosquitoes. *Sci. Rep.* **9**, 5340 (2019).
4. Huang, Y., Niu, B., Gao, Y., Fu, L. & Li, W. CD-HIT Suite: a web server for clustering and comparing biological sequences. *Bioinformatics* **26**, 680-682 (2010).
5. Nagai, H.*, et al.* Different role of IL-4 in the onset of hapten-induced contact hypersensitivity in BALB/c and C57BL/6 mice. *Br. J. Pharmacol.* **129**, 299-306 (2000).
