# Supplementary material for: ADP binding by the Culex quinquefasciatus mosquito D7 salivary protein enhances blood feeding on mammals
Source: Nat Commun. 2020 Jun 9;11:2911. doi: 10.1038/s41467-020-16665-z (PMC7283271; doi:10.1038/s41467-020-16665-z)
Supplement: Supplementary file 3 — Reporting Summary [file 41467_2020_16665_MOESM3_ESM.pdf]

nature research

Corresponding author(s): Eric Calvo

Last updated by author(s): 04/28/2020

Reporting Summary

Nature Research wishes to improve the reproducibility of the work that we publish. This form provides structure for consistency and transparency in reporting. For further information on Nature Research policies, see Authors & Referees and the Editorial Policy Checklist .

Statistics

For all statistical analyses, confirm that the following items are present in the figure legend, table legend, main text, or Methods section.

n/a	Confirmed	
☐	☒	The exact sample size (*n*) for each experimental group/condition, given as a discrete number and unit of measurement
☐	☒	A statement on whether measurements were taken from distinct samples or whether the same sample was measured repeatedly
☐	☒	The statistical test(s) used AND whether they are one- or two-sided<br>*Only common tests should be described solely by name; describe more complex techniques in the Methods section.*
☐	☒	A description of all covariates tested
☐	☒	A description of any assumptions or corrections, such as tests of normality and adjustment for multiple comparisons
☐	☒	A full description of the statistical parameters including central tendency (e.g. means) or other basic estimates (e.g. regression coefficient) AND variation (e.g. standard deviation) or associated estimates of uncertainty (e.g. confidence intervals)
☐	☒	For null hypothesis testing, the test statistic (e.g. *F*, *t*, *r*) with confidence intervals, effect sizes, degrees of freedom and *P* value noted<br>*Give P values as exact values whenever suitable.*
☒	☐	For Bayesian analysis, information on the choice of priors and Markov chain Monte Carlo settings
☒	☐	For hierarchical and complex designs, identification of the appropriate level for tests and full reporting of outcomes
☒	☐	Estimates of effect sizes (e.g. Cohen's *d*, Pearson's *r*), indicating how they were calculated

Our web collection on statistics for biologists contains articles on many of the points above.

Software and code

Policy information about availability of computer code

Data collection	qPCR data collected in CFX96 thermocycler, BioRad.<br>Protein chromatograms acquired in AKTA purifier (GE HealthCare).<br>Leica Confocal SP8 microscope for image acquisition.<br>Isothermal titration calorimetry data acquired in Microcal VP-ITC (Malvern).<br>Crystal data collected at the Advanced Photon Source, Argonne National Laboratory equipped with 10Hz Rayonix MX300HS detector.<br>Platelet aggregation data collected in a Chrono-Log Corporation aggregometer.<br>Absorbance data collected using a VersaMax microplate reader (Molecular Devices) and a Cytation 5 image reader (Biotek).<br>Mass spectrometry data collected in Orbitrap Fusion mass spectrometer (ThermoFisher Scientific).
Data analysis	CFX Maestro software version 1.1 (BioRad) for qPCR analysis.<br>Unicorn software version 5.3.1 (GE HealthCare) for HPLC chromatogram analysis.<br>Imaris software version 9.2.1 data analysis and Fiji ImageJ version 1.52n for post processing of immunofluorescence images.<br>Isothermal titration calorimetry data analyzed Microcal Origin software version 7 (OriginLab).<br>Crystal structure analysis done using Phaser version 2.8.3, Coot version 0.8.9.2, Phenix version 1.17.1-3660, PyMOL version 1.7.4, and UCSF Chimera version 1.13.1.<br>Graphs were prepared using GraphPad Prism software version 8.02.<br>PEAKS v10 (Bioinformatics Solutions Inc) for Mass spectrometry analysis.<br>CD-HIT software was used for clustering amino acid sequences for protein alignments.<br>Clustal Omega software (EMBL) was used for multiple protein alignments.<br>BoxShade server version 3.1 (Expasy Bioinformatics Resource Portal) was used for refining protein alignments.

For manuscripts utilizing custom algorithms or software that are central to the research but not yet described in published literature, software must be made available to editors/reviewers. We strongly encourage code deposition in a community repository (e.g. GitHub). See the Nature Research guidelines for submitting code & software for further information.

Data

Policy information about availability of data

All manuscripts must include a data availability statement. This statement should provide the following information, where applicable:

- Accession codes, unique identifiers, or web links for publicly available datasets
- A list of figures that have associated raw data
- A description of any restrictions on data availability

The source data underlying Figures 1a, 1b, 1c, 6a, 6b, 6c, 7a, 7b, and Supplementary Figures 2, 3b, 3c, 6, 7a, 7b, 7c, 7d 1c, 2b, 4c, 5a, 7c, 7d, and 8 are provided as a Source Data file. The datasets generated and/or analyzed during the current study are available from the corresponding author on reasonable request. The coordinates and structure factors of the crystal structure of CxD7L1 in complex with ADP have been deposited in the Protein Data Bank under the accession number 6V4C (https://www.rcsb.org/). Data currently on hold. It will be released by the Protein Data Bank upon article publication. Databases used in the study are as follows: NCBInr proteome (available at www.ncbi.nlm.nih.gov), the common Repository of Adventitious Proteins: cRAP.fasta database (available at ftp://ftp.thegpm.org/fasta/cRAP), PDB (https://www.rcsb.org/).

Field-specific reporting

Please select the one below that is the best fit for your research. If you are not sure, read the appropriate sections before making your selection.

[x] Life sciences [ ] Behavioural & social sciences [ ] Ecological, evolutionary & environmental sciences

For a reference copy of the document with all sections, see nature.com/documents/nr-reporting-summary-flat.pdf

Life sciences study design

All studies must disclose on these points even when the disclosure is negative.

Sample size	No statistical method were used to pre-determine the sample size. Samples sizes were selected based on previous experience to obtain reproducibility. For gene expression analysis, each sample consisted of 10 specimens, two biological and two technical replicates were analyzed in a similar manner as in Ribeiro et al. PLoS One 11, e0151400 (2016). For protein quantification in the salivary gland and saliva, 15 samples of individual salivary glands and three biological triplicates of saliva collected from 5 mosquitoes were used. For immunofluorescence experiments, 1 to 2 glands were imaged per experimental group of the salivary glands per experiment. For binding and platelet aggregation at least two experiments were assayed. For ADP determination, biological and technical duplicates were used. For tail vein bleeding assay, o 6 animals were used per group, expect group 2.5 μM of protein CxD7L2 that had 5 animals. For mass spectrometry, 3 biological replicates were analyzed independently.
Data exclusions	No data were excluded from the analyses.
Replication	Experimental findings were reliably reproduced in multiple independent experiments as indicated throughout the manuscript. qPCR study was performed twice. Protein quantification was performed in one experiment with a large number of samples. Western blots were repeated two times with different batches of proteins and antibody preparations giving consistent results. For immunofluorescence experiments, four independent experiments were performed. For ITC and platelet aggregation studies, measurements were repeated at least twice giving similar binding parameters. For ADP determination, one experiment was performed. For tail vein bleeding assay, one experiment with 41 mice was done. Mass spectrometry data were performed once in triplicates
Randomization	Salivary gland samples were randomly distributed for immunolocalization experiments. For tail vein bleeding assay, all animals used in the study were were pooled from different cages and were randomly distributed into control and experimental groups. For the rest of the experiments randomization does not apply.
Blinding	The investigators were not blinded to the allocation during the experiments or to the outcome assessment. The data presented did not require the use of blinding.

Reporting for specific materials, systems and methods

We require information from authors about some types of materials, experimental systems and methods used in many studies. Here, indicate whether each material, system or method listed is relevant to your study. If you are not sure if a list item applies to your research, read the appropriate section before selecting a response.

Materials & experimental systems

n/a	Involved in the study
☐	☒ Antibodies
☐	☒ Eukaryotic cell lines
☒	☐ Palaeontology
☐	☒ Animals and other organisms
☐	☒ Human research participants
☒	☐ Clinical data

Methods

n/a	Involved in the study
☒	☐ ChIP-seq
☒	☐ Flow cytometry
☒	☐ MRI-based neuroimaging

Antibodies

Antibodies used

For Western blot and immunolocalization of CxD7 proteins, antibodies either against Culex quinquefasciatus salivary gland extract, CxD7L1, or CxD7L2 were raised in rabbits in Noble Life Science facility according to their standard protocol (https://noblelifesci.com/custom-polyclonal-antibodies/), as described in methods section.
For ELISA and Western Blot, goat anti-rabbit conjugated to alkaline phosphatase (Sigma Cat # A3687). For immunofluorescence and confocal imaging, anti-rabbit IgG Alexa Fluor 594 (Thermo Fisher Cat # A11012) and Phalloidin Alexa 488 (Invitrogen cat. num.: A12379) were used.

Validation

The antibody validation of anti-rabbit conjugated to alkaline phosphatase, anti-rabbit IgG Alexa Fluor 594 , and Phalloidin Alexa 488 are provided on supplier websites:
https://www.sigmaaldrich.com/catalog/product/sigma/a3687?lang=en&region=US
https://www.thermofisher.com/antibody/product/Goat-anti-Rabbit-IgG-H-L-Cross-Adsorbed-Secondary-Antibody-Polyclonal/A-11012.
https://www.thermofisher.com/order/catalog/product/A12379#/A12379

For the validation of our primary antibodies raised in rabbits (anti-CxD7L1 and anti-CxD7L2 IgG) three dilutions were tested: 10 µg/mL, 1 µg/mL, and 0.5 µg/mL. For standarization of the ELISA for the protein quantification, the primary antibodies were tested at 10 µg/mL, 1 µg/mL, 0.5 µg/mL, and 0.1 µg/mL.

Eukaryotic cell lines

Policy information about cell lines

Cell line source(s)

Human embryonic kidney cells (HEK293) were purchased from ATCC (Cat# CRL-10852)

Authentication

It has been authenticated using STR by ATCC.

Mycoplasma contamination

The cell line was not tested for Mycoplasma contamination.

Commonly misidentified lines
(See ICLAC register)

No commonly misidentified cell line was used.

Animals and other organisms

Policy information about studies involving animals; ARRIVE guidelines recommended for reporting animal research

Laboratory animals

16 to 20-week-old female Balb/c mice were kept at 72 ± 3 °F, 30-70% humidity and a light/dark cycle of 14 hours of light and 10 hours of dark.
Culex quinquefasciatus mosquitoes were used in this study. Male and female adult mosquitoes were used for gene expression analysis (0-2 days old). For salivary gland collection, 4 to 7-day old female adult mosquitoes were used.

Wild animals

The study did not involve wild animals.

Field-collected samples

The study did not involve field-collected samples.

Ethics oversight

Public Health Service Animal Welfare Assurance #A4149-01 guidelines were followed according to the National Institute of Allergy and Infectious Diseases (NIAID), National Institutes of Health (NIH) Animal Office of Animal Care and Use (OACU). These studies were carried out according to the NIAID-NIH animal study protocols (ASP) approved by the NIH Office of Animal Care and Use Committee (OACUC), with approvals ID ASP-LMVR3 and ASP-LMVR102.

Note that full information on the approval of the study protocol must also be provided in the manuscript.

Human research participants

Policy information about studies involving human research participants

Population characteristics	Healthy donors, males or females of unknown age that had not taken antiinflamatory drugs or aspirin for a week before the blood donation.
Recruitment	Research blood donors provide written informed consent, and platelets were de-identified prior to distribution.
Ethics oversight	National Cancer Institute Institutional Review Board (NCI IRB) approved NIH protocol 99-CC-0168, "Collection and Distribution of Blood Components from Healthy Donors for In Vitro Research Use."

Note that full information on the approval of the study protocol must also be provided in the manuscript.
